# Modeling the spatial distribution of anthrax in southern Kenya

**DOI:** 10.1371/journal.pntd.0009301

**Published:** 2021-03-29

**Authors:** Fredrick Tom Otieno, John Gachohi, Peter Gikuma-Njuru, Patrick Kariuki, Harry Oyas, Samuel A. Canfield, Jason K. Blackburn, M. Kariuki Njenga, Bernard Bett

**Affiliations:** 1 Animal Health Program, International Livestock Research Institute, Nairobi, Kenya; 2 Department of Environmental Science and Land Resources Management, School of Environment, Water and Natural Resources, South Eastern Kenya University, Kitui, Kenya; 3 Washington State University, Global Health Kenya, Nairobi, Kenya; 4 School of Public Health, Jomo Kenyatta University of Agriculture and Technology, Nairobi, Kenya; 5 Veterinary Epidemiology and Economics Unit, Kenya Ministry of Agriculture, livestock and Fisheries, Nairobi, Kenya; 6 Spatial Epidemiology and Ecology Research Laboratory, Department of Geography, University of Florida, Gainesville, Florida, United States of America; 7 Emerging Pathogens Institute, University of Florida, Gainesville, Florida, United States of America; Faculty of Medicine and Health Sciences, Universiti Putra Malaysia, MALAYSIA

## Abstract

**Background:**

Anthrax is an important zoonotic disease in Kenya associated with high animal and public health burden and widespread socio-economic impacts. The disease occurs in sporadic outbreaks that involve livestock, wildlife, and humans, but knowledge on factors that affect the geographic distribution of these outbreaks is limited, challenging public health intervention planning.

**Methods:**

Anthrax surveillance data reported in southern Kenya from 2011 to 2017 were modeled using a boosted regression trees (BRT) framework. An ensemble of 100 BRT experiments was developed using a variable set of 18 environmental covariates and 69 unique anthrax locations. Model performance was evaluated using AUC (area under the curve) ROC (receiver operating characteristics) curves.

**Results:**

Cattle density, rainfall of wettest month, soil clay content, soil pH, soil organic carbon, length of longest dry season, vegetation index, temperature seasonality, in order, were identified as key variables for predicting environmental suitability for anthrax in the region. BRTs performed well with a mean AUC of 0.8. Areas highly suitable for anthrax were predicted predominantly in the southwestern region around the shared Kenya-Tanzania border and a belt through the regions and highlands in central Kenya. These suitable regions extend westwards to cover large areas in western highlands and the western regions around Lake Victoria and bordering Uganda. The entire eastern and lower-eastern regions towards the coastal region were predicted to have lower suitability for anthrax.

**Conclusion:**

These modeling efforts identified areas of anthrax suitability across southern Kenya, including high and medium agricultural potential regions and wildlife parks, important for tourism and foreign exchange. These predictions are useful for policy makers in designing targeted surveillance and/or control interventions in Kenya.

We thank the staff of Directorate of Veterinary Services under the Ministry of Agriculture, Livestock and Fisheries, for collecting and providing the anthrax historical occurrence data.

## Author summary

Anthrax is a neglected zoonosis worldwide. In Kenya, outbreaks have been reported in wildlife, livestock, and humans, resulting in severe public health burden and socio-economic impacts. Because of this, anthrax is ranked as the highest priority disease in the country. To identify factors that influence the spatial distribution of the disease in Kenya, we analyzed surveillance on available anthrax outbreaks recorded in the southern half of the country. Areas predicted to be highly suitable for the disease were predominantly in the southwestern region around the shared Kenya-Tanzania border running as a belt through central regions and central highlands of Kenya. These suitability regions extend westwards to cover large areas in western highlands and the western regions around Lake Victoria and bordering Uganda. The entire eastern and lower-eastern regions towards the coastal region were predicted to have lower suitability for anthrax. Cattle density, rainfall of wettest month, soil clay content, soil pH, soil organic carbon, length of longest dry season, vegetation index and temperature seasonality were key variables predicting the distribution of anthrax in the region. The study generated a suitability map depicting geographical areas that can be targeted for risk-based surveillance and or control measures for the disease.

## Introduction

Anthrax is a zoonosis caused by the spore-forming bacterium *Bacillus anthracis* and is of global importance that mainly affects domestic and wild herbivores. Its occurrence patterns are influenced by environmental, socioeconomic and cultural factors [1,2]. In Kenya, anthrax is endemic and has been ranked as the most important zoonotic disease [3]. Impacts of anthrax outbreaks include reduced livestock production due to mortality and associated socio-economic losses, higher public health burden, and decimation of wildlife [4]. Analyses conducted in other regions have demonstrated environmental, socioeconomic, and cultural factors can structure anthrax occurrence patterns [1,2], and moreover, studies show that the disease clusters in specific ecological conditions [5,6]. However, there is limited knowledge on factors associated with the distribution of anthrax in Kenya. Such information is required to estimate the spatial distribution of the disease in the country and to target surveillance and control measures.

Ecological niche models (ENMs) aim to correlate species’ occurrence data (point locations) with environmental covariates (gridded data) to determine suitable environmental conditions that meet a species’ ecological requirements. Those requirements are then mapped onto the landscape to predict areas of relative habitat suitability [7,8]. The ENM predictions provide test for biogeographical hypothesis that spatial variation in the numbers and types of species results from interactions from the species and their environments [9]. ENMs have been used widely to map the potential geographic distribution of a wide range of taxa. ENMs utilize algorithms such as machine learning and rule-based decision trees [10–12] and they are often fitted to presence/absence or presence-only data to map potential distributions. Many machine learning and rule-based algorithms have been developed over time; these include boosted regression trees (BRT), random forests (RF), maximum entropy (Maxent), genetic algorithm for rule-set prediction (GARP), generalized additive models (GAMs) and generalized linear models (GLMs) among others [13–17]. They are increasingly being used to analyze disease surveillance records from government registries [12,18]. ENMs have been used to predict areas that are suitable for *B*. *anthracis* globally [19] and across several countries: Australia, USA and Mexico, China, Ghana, Italy, Kazakhstan, Kyrgyzstan, West Africa, Tanzania, and Zimbabwe [10,12,20–23]. Over the past 60 years, historical outbreak data have been recorded in Kenya. Here we use those data that had known geographical coordinates to model the potential geographic distribution of anthrax in Kenya.

The objective of this current study was to predict the potential geographic distribution of anthrax in Kenya using a BRT algorithm and to identify the main predictor variables influencing the distribution. BRTs have recently been considered a dominant algorithm for mapping transmission risk of infectious zoonoses [19,24]. Furthermore, several studies noted high predictive performance [25–27]. BRTs have been used to model the distribution of anthrax over several landscapes [19,21,26].

## Materials and methods

### Ethics statement

This study was a component of a broader research initiative on anthrax hotspots in Kenya where the approvals were obtained from KEMRI Scientific and Ethics Review Unit (SERU) (Ref: KEMRI/RES/7/3/1).

### Study area

**Fig 1:**
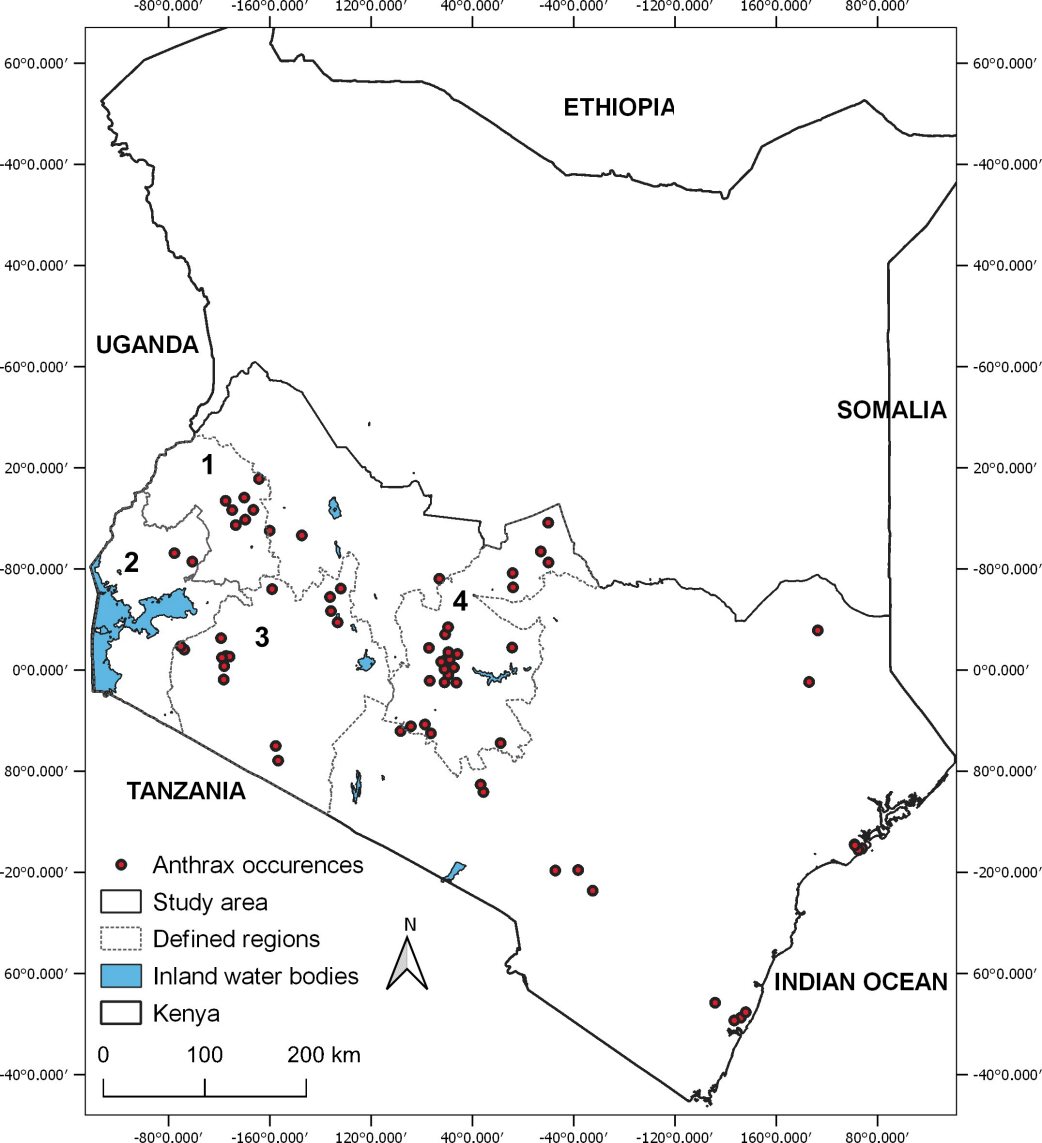
Map of Kenya showing the study area and the spatial distribution of anthrax occurence data (red circles) recorded between 2011 and 2017. Areas 1–4 arbitrarily represent important regions for describing the predicted distribution of anthrax: 1) western highlands; 2) Lake Victoria basin; 3) southwestern region; 4) central highlands.

This study was limited to the southern half of Kenya, as more than 95% of all reported outbreaks that could be mapped with certainty were within the study area [28]. The selected area extends between Latitude 4°40’38′′ S to 1°56′59′′ N Latitude and 33°56’28′′ E to 41°35′24′′ E Longitude (Fig 1) and represents ~50% of the country’s total land surface and ~91% of the Kenyan human population, with those below poverty line Headcount Index ranging between 10.3 and 84.9 (mean = 43.4) [29]. The mean cattle density in this area was estimated at 3666 Tropical Livestock Units (TLU) [30].

The study area encompasses all major wildlife protected areas, including Lake Nakuru National Park, which reported multiple anthrax outbreaks in the recent past [5]. Based on World Reference Base (WRB) classification [31] the types of soils found in the area were diverse (totalling 66 types). Soils with calcium carbonate covered 13% of the total area; these have been associated with anthrax endemic areas [32].

The climate in this study area ranges from humid tropical, along the coast to temperate and sub-tropical inland and hot and dry in arid and semi-arid areas in the mainland areas. The area has a bimodal seasonal pattern with the long rains season observed between March and June, and short rainy season between September and December. Mean temperatures generally vary with elevation although there has been increased variability in temperature in recent years, with estimated increase of 1.0°C since 1960. In addition, rainfall distribution has changed in space and time, but its intensity has largely remained the same [33,34].

## Data management and analysis

### Occurrence data and generation of pseudo-absence data

A total of 666 anthrax outbreak records collected between 1957 to 2017 were obtained from the Directorate of Veterinary Services (DVS) archives [6]. Out of these, 86 records of livestock outbreaks reported between 2011 to 2017 could be mapped to the geographic coordinates of the outbreak and were used as occurrence data. As this study applies a presence/absence modeling approach, occurrence data were thinned to a single point per pixel of the resolution of environmental covariates used [35,36]. For each modeling experiment (see below), an equal number of pseudo-absence points were randomly generated within the study area. All pseudo-absence points were at least 5km from each of the 69 spatially unique presence points to build unbiased and reliable models [37].

### Environmental data and variable selection

Climatic and other environmental data hypothesized to influence the spatial distribution of anthrax were downloaded from online databases; sources of these data are shown in (S1 Text). Covariates were downloaded as raster files, clipped to the study area, and resampled to 250m resolution with bilinear technique. Before fitting the BRT models, we used the variable inflation factor (VIF) to test for multicollinearity with cut-off of VIF < 10 to reduce highly correlated variables [34]. The VIF is a measure of the degree of multi-collinearity between independent variables in a regression model; where small VIF values indicate low correlation among variables while large VIF greater than 10 indicate severe collinearity [38] All data sets were processed and analyzed using QGIS 3.1.6.0 and R 3.5.3 [39,40].

### Model building and evaluation

Here we implemented a BRT algorithm. Briefly, BRTs combine the strengths of regression trees and boosting to build many simple decision trees adaptively. Thus, BRT combines statistical and machine learning methods to combine large numbers of shallow trees, improving prediction across the process. The BRT performance can be further improved by tuning several hyperparameters (values used to control the model learning process) detailed in [13]: bagging fraction (bf) introduces randomness into the model by defining the proportion of data drawn at random from the original data at each step, thereby improving performance and reducing overfitting; tree complexity (tr) defines the number of nodes for each tree; learning rate (lr) varies the contribution of each tree added to the model and defines the number of trees preferable under several observations and computational time available for model fitting i.e. smaller learning rate results in larger number of trees.

The BRTs were built using the ‘gbm’ package (‘gbm.step’ extension) in R 3.5.3 [41]. We employed a bootstrapping, or ensemble approach, generating 100 individual BRT experiments. For each experiment, new pseudo-absence data were generated and combined with the presence data. The combined data were then partitioned into model training (75% of the data) and model evaluation sets (25% of the data).

We assessed ‘gbm.step’ function settings to obtain the best predictive performance based on AUC under different bagging conditions, learning rate and tree complexity and the parameters chosen based on the minimum predictive error. The final ‘gbm.step’ was thus set to fit the training data with learning rate (lr) = 0.001, bagging fraction (br) = 5 and maximum tree = 2500. Model performance was evaluated using AUC (area under the curve) ROC (receiver operating characteristics) curves for each experiment and averaged across all experiments. AUC has been identified as the most prominent among methods of evaluating ENMs ability to predict the observed distributions [28], however, its reliability has also been challenged [42]. Predictions for each experiment (n = 100) were generated and averaged to obtain a final anthrax distribution map for the study area; the lower 2.5% and upper 97.5% confidence intervals were also mapped.

In order to gain more insights into the model predictions, partial dependency plots (PDPs) were generated. The PDPs graphically illustrate the functional relationship between the target response and the set of predictors [43]. The PDPs were generated with pdp R package [43] for each and across experiments to demonstrate how each individual predictor influenced mean prediction probabilities and strength of its contribution to the prediction.

## Results

### Variable selection

VIF analyses filtered the 41 candidate variables (S1 Text) to 18 independent variables (Table 1). These 18 independent variables were fitted in the modeling process.

**Table 1:** Variables fitted in BRT algorithm for niche modeling.

	Variables	Units
**1.**	Rainfall wettest month	mm
**2.**	Temperature Seasonality	°c*10
**3.**	Calcic Vertisols	%
**4.**	Soil organic carbon density	kg/m3
**5.**	Clay content	mass fraction (%)
**6.**	Cattle density	animals/km2
**7.**	Enhanced vegetation index	index
**8.**	Haplic Calcisols	%
**9.**	Haplic Vertisols	%
**10.**	Annual Average Relative Humidity	%
**11.**	Length of longest dry season	months
**12.**	Palmer Drought Severity Index	index
**13.**	Potential evapotranspiration	mm
**14.**	Soil pH	pH
**15.**	Silt content	mass fraction (%)
**16.**	Slope	degrees
**17.**	Soil Moisture	m^3/m^3
**18.**	Soil texture	factor

### Predicted distribution of anthrax suitability

**Fig 2:**
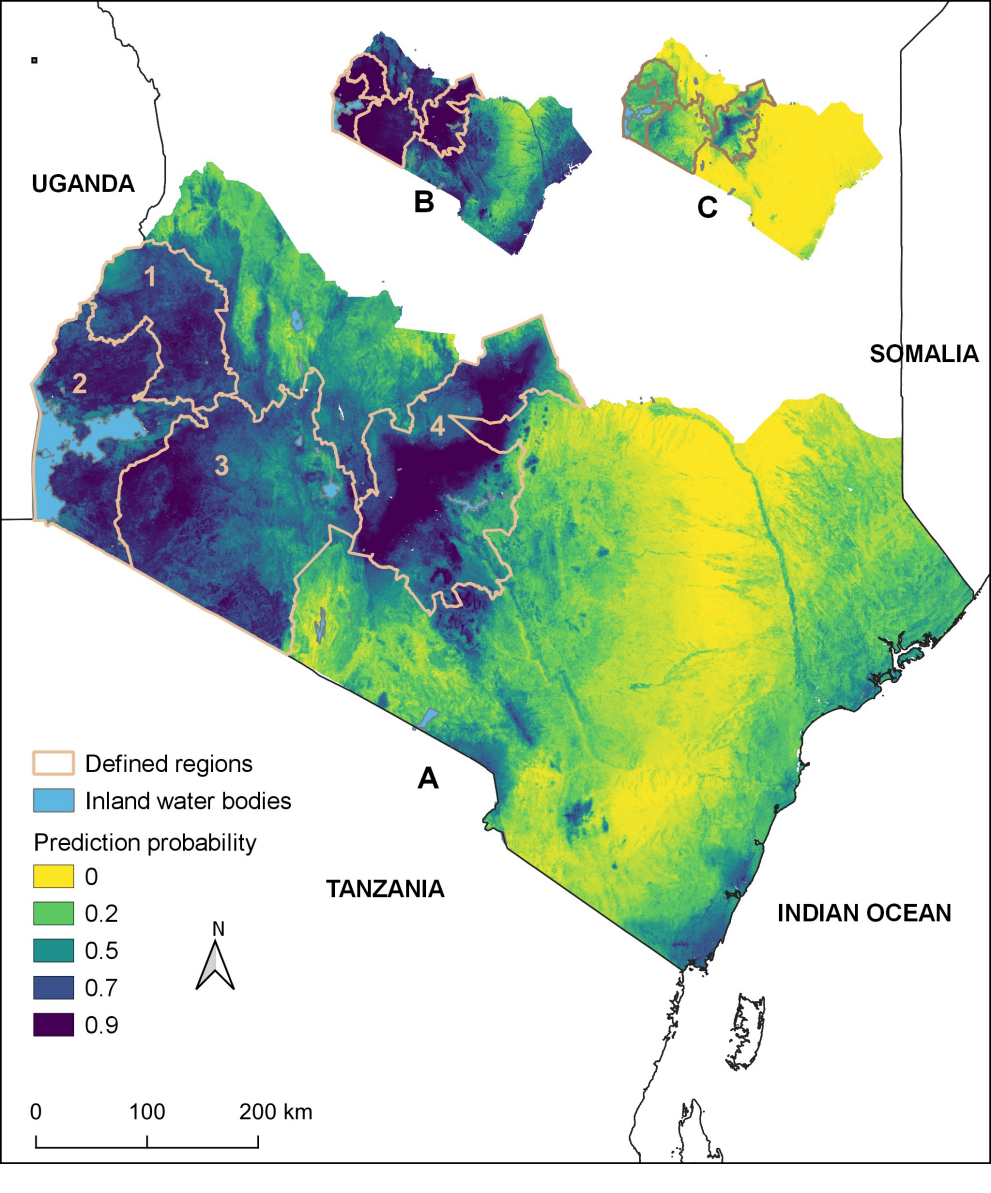
Panel A: Predicted geographic distribution of anthrax in southern Kenya based on the mean prediction of an ensemble of 100 boosted regression tree experiments. **Panel B shows the upper 97.5% and C the lower 2.5% confidence intervals.** Regions 1–4 are used as reference areas for the discussion: 1) western highlands; 2) Lake Victoria basin; 3) southwestern region; 4) central highlands.

**Fig 3:**
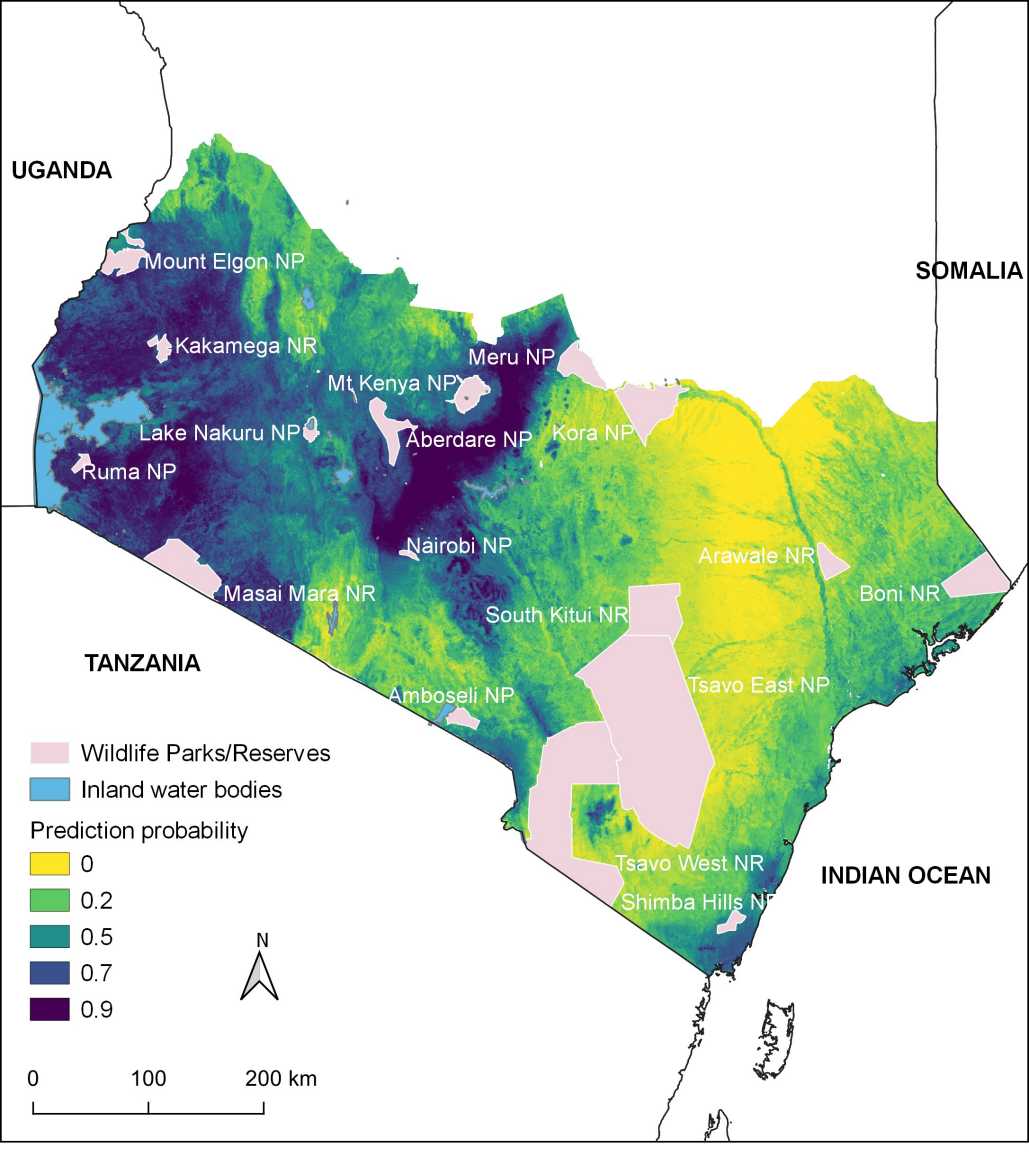
Location of National Parks and Game Reserves overlaid on the predicted distribution of anthrax in southern Kenya based on boosted regression tree experiments.

Fig 2 illustrates the potential distribution of anthrax for southern Kenya. The mean AUC of the ensemble was 0.8. The proportion of the study area predicted to be suitable for anthrax at probability > 0.6 was 22% of the study area. These areas were predominantly in the defined regions (1–4). Areas to the periphery of these regions and along the coastal strip had suitability probabilities ranging between 0.4 and 0.6. The entire eastern and lower-eastern regions towards the coastal region were predicted to have lower suitability (probability of <0.2) for anthrax. The predicted high suitability areas included areas near wildlife national parks and game reserves including Nairobi, Nakuru, Mount Kenya, Mwea Mount Elgon National Parks, and Masai Mara National Reserve (Fig 3).

**Fig 4:**
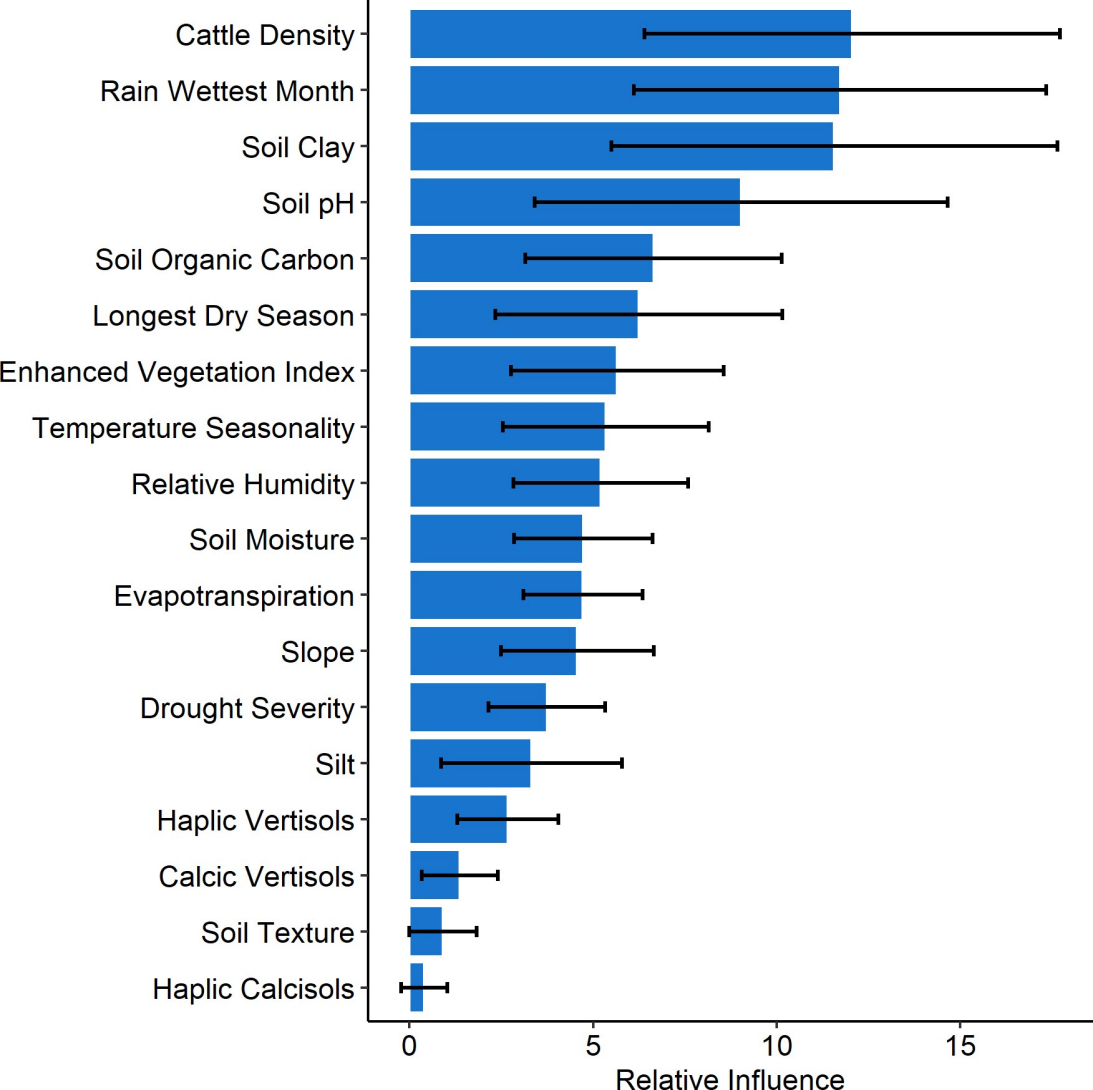
Variable relative influence for final variable set used to model the distribution of anthrax in southern Kenya using boosted regression tree experiments. Error bars represent variability across an ensemble of 100 BRT experiments.

Relative variable influence across the 100 BRT experiments is illustrated in Fig 4. Cattle density, rainfall of wettest month, soil clay content, soil pH, soil organic carbon, length of longest dry season, vegetation index, and temperature seasonality were more important across the experiments.

**Fig 5:**
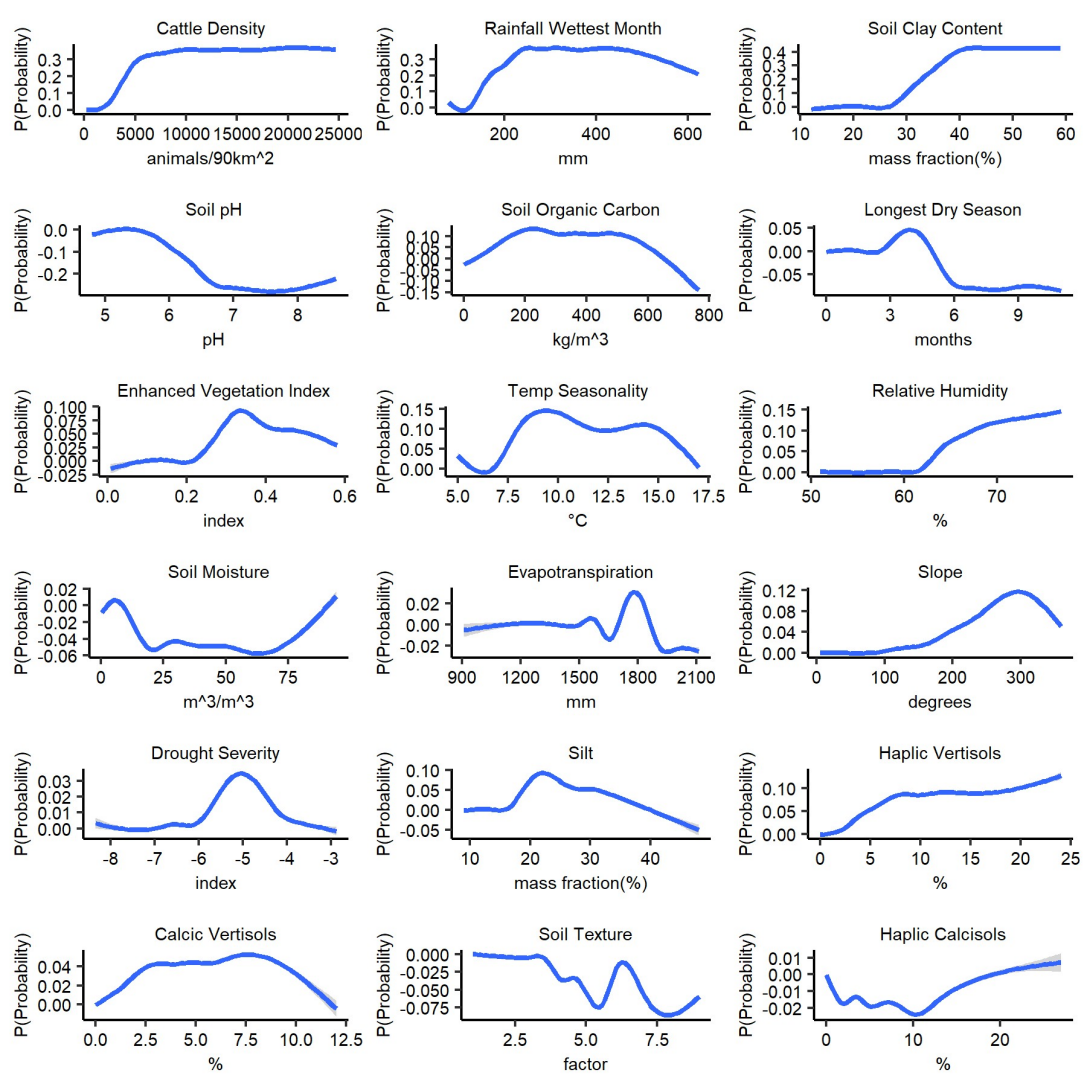
Partial dependency plots (PDP) showing marginal effects on the mean prediction probability of potential anthrax distribution by each variable across the 100 BRT experiments.

Partial dependency plots (PDP) are illustrated in Fig 5. High cattle density, increased rainfall of the wettest month (between ~200–500 mm), and high percentage of soil clay content (~35–45%) were associated with high anthrax probability. A moderate enhanced vegetation index (associate with grasslands) was also predictive. These variables have been hypothesized as important for predicting anthrax in other ENM studies.

## Discussion

This study predicted the geographic distribution of anthrax in southern Kenya using livestock surveillance records collected by the Directorate of Veterinary Services (DVS) between 2011 and 2017 and publicly available environmental data using a boosted regression trees ensemble modeling approach. Our model predicted areas with environmental conditions suitable for anthrax predominantly in the southwestern regions around the shared Kenya-Tanzania border and running as a belt through central highlands of Kenya. These suitable regions extend westwards to cover large areas in western highlands and regions around Lake Victoria bordering Uganda. However, the proportion of these suitable, particularly those predicted with suitability probability of > 0.6 was 22% of the total study landmass (~580,367km^2^). The entire eastern, and lower-eastern regions towards the coastal region were predicted to have lower suitability. Variables found to have the greatest contribution to the potential distribution of anthrax comprised of (a) soil properties—clay content, pH and organic carbon; (b) climatic variables—rainfall of wettest month, temperature seasonality and length of longest dry season (c) cattle demography—cattle density; and (d) environmental variable—vegetation index.

The regions that our models predicted to be suitable for anthrax are largely characterized by high and medium agricultural potential with established history of intensive and extensive livestock production including mixed crop-livestock farming systems in Kenya. These regions include substantial areas used by colonial settlers for beef and dairy agriculture and termed “white highlands”. Consistent with previous predictions, human and livestock anthrax vulnerability is concentrated in rural rain-fed systems similar to other predictions [19]. Livestock movements through trade may have disseminated *Bacillus anthracis*, the causative agent of anthrax, across suitable regions impacting the distribution defined by occurrence records. These regions also handle intense human activities characterized with inadequate knowledge on anthrax carcass disposal, a pathway for inter-region dispersion and local persistence of *B*. *anthracis*, perhaps influencing the geographical distribution of the disease as well. We estimate that the human population in the predicted suitable areas on a probability of >0.6 is 27,988,699 based on World spatial population 2020 [44]; the majority facing well-known anthrax occupational exposure as animal health practitioners, farmers, herders, butchers and meat sellers.

Interestingly, despite our study employing livestock anthrax occurrences alone, our model predicted regions suitability for anthrax in wildlife conservation areas that our team has previously reported as likely anthrax hotspots such as Nakuru National Park [4,5]. Nevertheless, it is known that the entire periphery and rarely the interior of wildlife conservation areas are generally shared by both livestock and wildlife, presenting possible bidirectional anthrax transmission interfaces such as those hypothesized for bovine tuberculosis or foot-and-mouth disease (FMD) [45,46]. Indeed, the model predicted likely transboundary anthrax suitability across the larger Mara-Serengeti ecosystem along the shared Kenya-Tanzanian border. Previous studies have reported anthrax occurrences in Serengeti National Park in Tanzania with prediction maps similar to ours identifying areas suitable along the border on the Tanzanian side [47,48]. If not curbed, anthrax could impede wildlife conservation efforts, particularly for endangered species inhabiting these anthrax-suitable ecosystems. Challenges in reporting anthrax outbreaks in wildlife include underestimation of anthrax burden in wildlife. Niche modeling, such as carried out in this study, presents opportunities for a better approximation of geographical risk. For instance, the lower eastern regions towards the coastal region, home to the vast Tsavo National Park, were predicted as lowly suitable for anthrax reflecting previous findings [5].

*Bacillus anthracis* is an environmental pathogen. Therefore, the occurrence and distribution of anthrax is expected to be limited by various climatic variables. Thus specific patterns of rainfall, temperature, and their seasonality have been applied to determine anthrax distribution in previous niche modeling studies [10,49,50]. In our study, precipitation level of the wettest month and temperature seasonality were predicted to influence anthrax distribution similar to studies in Kazakhstan and South Africa, respectively [18,51]. Precipitation provides water that may influence anthrax in a number of ways–exposing buried spores to the surface, collecting and concentrating spores in ‘storage areas’ and possibly dispersing the spores through water run-off [52]. Length of dry season is also suggested to be associated with anthrax outbreaks similar to a previous study in Tanzania [49]. Animals feeding on short grass close to the soil during dry season are more exposed to spores increasing chances of anthrax outbreaks [53]. The dry season also leads to water and forage scarcity precipitating a likely livestock-wildlife anthrax transmission interfaces, at grazing grounds and water points [54].

Soils with high calcium concentrations and a pH > 6.1 are known to influence the global distribution of anthrax through enabling spore germination, growth, survival, and possibly re-sporulation in the soil [55]. Also, soil clay content and pH, contained in Vertisol soils are reported to have a direct influence on germination and sporulation of *B*. *anthracis* [19,56]. In our study, soil clay was positively associated with anthrax prediction, similar to a recent study in Minnesota, USA [57]. Additionally, soil clay may play an indirect role by accelerating flooding due to their high water-retaining capacity, concentrating the spores, and later providing conducive environments for the growth of contaminated herbage that attracts grazing by livestock [58]. Our study findings suggest that the regions predicted as suitable for anthrax may contain adequate soil pH to maintain bacterial spores. Having predicted regions suitable for anthrax and the identified environmental variables, it is possible to design more focused studies to elucidate the mechanisms behind the possible long-term survival of anthrax spores and outbreak occurrence in the regions.

Apart from our study picking environmental variables as important, which can be attributed to environmental conditions defining *B*. *anthracis* niches, our study also found cattle density to be positively associated with anthrax distribution similar to studies in China and countries located in the Northern Hemisphere [11,21]. High cattle density presents a greater likelihood of exposure from shared contaminated grazing and/or watering points per capita, as sometimes seen in wildlife [59].

These results generated a practical and actionable map for targeting anthrax surveillance and control in livestock areas and nearby wildlife management or conservation areas. Sustained annual livestock vaccination campaigns remain the best-bet method for anthrax control in both humans and livestock. Data from Azerbaijan confirm that anthrax control in livestock has measurable reduction in human disease burden [60]. However, the vaccine must be administered to livestock annually to reduce disease occurrence. A first step in implementing vaccination is to identify priority areas for targeting campaigns. However, the vaccine is administered in injections and therefore is not practical to use in wildlife [60,61]. Surveillance therefore remains critical in wildlife management or protected area, especially areas where livestock and wildlife may commingle. The map generated in this study can be used to prioritize surveillance sites in the parks factoring in the logistical challenges in these locations.

This study had several limitations. The occurrences data used in our study gave a small sample size which can be associated with sampling bias [62]. However, ENM modelling approaches are robust enabling use of few and/or biased occurrence data. Broadly, BRTs and ENMs can perform well with small sample sizes [63]. High model accuracy has been observed for models based on sample size as small as 5, 10 and 25 relative to models of 100 samples [64]. The same study determined that model performance depends on both sample size and species’ prevalence and increases with decreasing prevalence under constant sample size. In our study, we restricted modelling to a small area of southern Kenya which contained 95% of all the anthrax occurrence data decreasing prevalence and reducing bias, both of which increased model performance. By limiting the study to the southern half of Kenya, where the occurrence data were concentrated, interpretations for the entire country was also limited. However, a more intensive anthrax surveillance system has been established within our team to obtain more representative occurrence data at the national level, to extend our analyses and predictions to the national level in the future. The resolution of some of the spatial data sets (e.g., climate data) is not granular enough. Their refinement is also hampered by the poor distribution of synoptic meteorological stations that could provide primary data for correcting these data. AUC accuracy metrics which we applied for model evaluation have been criticized as not optimal for ENM models accuracy evaluation [42,65], but novel approaches are being explored.

In conclusion, our study predicted areas likely to be anthrax-prone, serving as a proxy of anthrax risk with associated variables in southern Kenya. These findings covering southern counties can be implemented in policy, decision support, and protecting public health at the county level through a One Health approach. Results can then be projected to the whole of Kenya and tested with incoming surveillance data. The findings will also inform future ecological and epidemiological research.

## Supporting information

S1 Text Candidate variable descriptions, online sources and references. (DOCX)
