# Supplementary material for: Modeling the spatial distribution of anthrax in southern Kenya
Source: PLoS Negl Trop Dis. 2021 Mar 29;15(3):e0009301. doi: 10.1371/journal.pntd.0009301 (PMC8032196; doi:10.1371/journal.pntd.0009301)
Supplement: S1 Text — (DOCX) [file pntd.0009301.s001.docx]

**S1 Text. Data description, online source and references**

Candidate predictor variables descriptions

|  | **Variable (source)** | **Units** |
| --- | --- | --- |
| 1 | 30-arc seconds Mean annual AET (Trabucco *etal,* figshare) | mm |
| 2 | 0.5° resolution Relative Humidity (CRU) | % |
| 3 | Slope (derived from GOTOPO30) | degrees |
| 4 | 1km resolution Mean annual temperature (University of York, AfriClim) | ⁰C*10 |
| 5 | 1km resolution Mean temp warmest quarter (University of York, AfriClim) | ⁰C*10 |
| 6 | 1km resolution Mean temp coolest quarter (University of York, AfriClim) | ⁰C*10 |
| 7 | 1km resolution Mean annual rainfall (University of York, AfriClim) | mm |
| 8 | 1km resolution Rainfall wettest month (University of York, AfriClim) | mm |
| 9 | 1km resolution Precipitation of Driest Month (University of York, AfriClim) | mm |
| 10 | 1km resolution Rainfall seasonality (University of York, AfriClim) | mm |
| 11 | 1km resolution Rainfall wettest quarter (University of York, AfriClim) | mm |
| 12 | 1km resolution Rainfall driest quarter (University of York, AfriClim) | mm |
| 13 | 1km resolution Mean diurnal range in temp (University of York, AfriClim) | ⁰C*10 |
| 14 | 1km resolution Isothermality (University of York, AfriClim) | ⁰C*10 |
| 15 | 1km resolution Temperature Seasonality (University of York, AfriClim) | ⁰C*10 |
| 16 | 1km resolution Max temp warmest month (University of York, AfriClim) | ⁰C*10 |
| 17 | 1km resolution Min temp coolest month (University of York, AfriClim) | ⁰C*10 |
| 18 | 1km resolution Annual temperature range (University of York, AfriClim) | ⁰C*10 |
| 19 | 250m resolution Soil organic carbon density (depth 0 cm) (ISRIC) | kg/m3 |
| 20 | 5 arc-minute Gridded Livestock density (Harvard Dataverse) | animals per km2 |
| 21 | 0.05° resolution Climate Hazards Infrared Precipitation (Climate Hazard Group) | total mm/year |
| 22 | 250m resolution Clay content (0-2 micrometer) at depth 0.00 m (ISRIC) | mass fraction (%) |
| 23 | 1km resolution Number of dry months (University of York, AfriClim) | months |
| 24 | 250 m resolution Enhanced vegetation index (**AfSIS**) | index |
| 25 | 1km resolution Length of longest dry season (University of York, AfriClim) | months |
| 26 | 1km resolution Annual moisture index (University of York, AfriClim) | index |
| 27 | 1km resolution Moisture index moist quarter (University of York, AfriClim) | index |
| 28 | 1km resolution Moisture index arid quarter | index |
| 29 | 4 km resolution Palmer Drought Severity Index (TerraClimate) | index |
| 30 | 30 arc seconds Potential evapotranspiration (University of York, AfriClim | mm |
| 31 | 250 m resolution Soil texture fraction at depth 0.00 m | factor |
| 32 | 4 km resolution Soil Moisture (TerraClimate) | m^3/m^3 |
| 33 | 30-arc seconds digital elevation model (USGS) | meters |
| 34 | 30 arc seconds. Priestley-Taylor Alpha Coefficient Soil-Water Balance (Trabucco *etal,* figshare) | P-Tα |
| 35 | 4 km resolution Runoff (TerraClimate) | mm per yr |
| 36 | 250 m resolution Silt content (2-50 micrometer) at depth 0.00 m (ISRIC) | mass fraction % |
| 37 | 250m resolution sand content (50-2000 micrometer) depth 0.00m | mass fraction (%) |
| 38 | 250 m resolution Soil pH x 10 in H2O at depth 0.00 m (ISRIC) | Index*10 |
| 39 | 250 m resolution Calcic Vertisols WRB class (ISRIC) | % |
| 40 | 250 m resolution Haplic Vertisols WRB class (ISRIC) | % |
| 41 | 250 m resolution Haplic Calcisols (ISRIC) WRB class (ISRIC) | % |

Data sources and reference

| Data | Source | Reference |
| --- | --- | --- |
| Bioclimatic (temperature; precipitation; seasonal variables) | **University of York, AfriClim:**  https://webfiles.york.ac.uk/KITE/AfriClim/GeoTIFF_30s/baseline_worldclim/ | [6] |
| Climate hazards infrared precipitation (CHIRPS) | **Climate Hazard Center:**  https://data.chc.ucsb.edu/products/CHIRPS-2.0/ | [3] |
| Actual evapotranspiration; soil-water balance | **Trabucco *etal,* figshare:**  https://figshare.com/articles/Global_High-Resolution_Soil-Water_Balance/7707605/3 | [7] |
| Drought severity index; runoff; soil moisture | **TerraClimate:**  https://climate.northwestknowledge.net/TERRACLIMATE/index_directDownloads.php | [1] |
| Enhanced vegetation index | **AfSIS:**  http://africasoils.net/services/data/remote-sensing/land | [2] |
| Soil type, Soil properties | **ISRIC:**  https://www.isric.org/explore/soilgrids | [5] |
| Relative humidity | **CRU:**  https://crudata.uea.ac.uk/cru/data/hrg/tmc/ | [6] |
| Elevation [GOTOPO30) | **USGS:**  https://earthexplorer.usgs.gov/ | [7] |
| Cattle density | **HAVARD, Dataverse:**  https://dataverse.harvard.edu/dataset.xhtml?persistentId=doi:10.7910/DVN/GIVQ75 | [4] |
| Slope | Derived from Elevation (GOTOPO30) |  |

REFERENCES

1. Abatzoglou JT, Dobrowski SZ, Parks SA, Hegewisch KC. TerraClimate, a high-resolution global dataset of monthly climate and climatic water balance from 1958-2015. In: Climatology Lab UoI, editor.: Climatology Lab, University of Idaho; 2018.
2. Didan K. MOD13Q1 MODIS/Terra vegetation indices 16-day L3 global 250m SIN grid V006. In: DAAC NELP, editor.: African Soil information Services; 2015.
3. Funk C, Peterson P, Landsfeld M, Pedreros D, Verdin J, Shukla S, et al. The climate hazards infrared precipitation with stations—a new environmental record for monitoring extremes. In: Center CH, editor. Hazards Infrared Precipitation (CHIRPS) University of California, Santa Barbara; 2015.
4. Gilbert M, Nicolas G, Cinardi G, Van Boeckel TP, Vanwambeke SO, Wint GW, et al. Global cattle distribution in 2010. V3 ed: Harvard Dataverse; 2018.
5. Hengl T, de Jesus JM, Heuvelink GB, Gonzalez MR, Kilibarda M, Blagotić A, et al. SoilGrids250m: Global gridded soil information based on machine learning. PLoS One. 2017;12(2):e0169748.
6. New M, Lister D, Hulme M, Makin I. A high-resolution data set of surface climate over global land areas. In: Climatic Research Unit UoEA, editor. A high-resolution data set of surface climate over global land areas: Climatic Research Unit; 2002.
7. Platts PJ, Omeny PA, Marchant R. AFRICLIM: high‐resolution climate projections for ecological applications in Africa. AFRICLIM: high‐resolution climate projections for ecological applications in AfricaThe University of York; 2015.
8. Trabucco A, Zomer R. Global soil water balance geospatial database. In: Information CCfS, editor. Global soil water balance geospatial database2010.
9. USGS. Global 30 Arc-Second Elevation (GTOPO30). In: USGS, editor. 1996.
